# Putative tumour-suppressor gene *DAB2 *is frequently down regulated by promoter hypermethylation in nasopharyngeal carcinoma

**DOI:** 10.1186/1471-2407-10-253

**Published:** 2010-06-03

**Authors:** Joanna H Tong, David C Ng, Shuk L Chau, Ken K So, Patrick P Leung, Tin L Lee, Raymond W Lung, Michael W Chan, Anthony W Chan, Kwok W Lo, Ka F To

**Affiliations:** 1 Department of Anatomical and Cellular Pathology, The Chinese University of Hong Kong, Hong Kong, PR China; 2 State Key Laboratory in Oncology in South China, Li Ka-Shing Institute of Health Sciences, The Chinese University of Hong Kong, Hong Kong, PR China; 3 Institute of Digestive Disease, The Chinese University of Hong Kong, Hong Kong, PR China; 4 The Eunice Kennedy Shriver National Institute of Child Health and Human Development, National Institutes of Health, Bethesda, USA; 5 Department of Life science, National Chung Cheng University, Min-Hsiung, Chia-Yi, Taiwan, Republic of China

## Abstract

**Background:**

Human Disabled-2 (DAB2), is a multi-function signalling molecule that it is frequently down-regulated in human cancers. We aimed to investigate the possible tumour suppressor effect of DAB2 in nasopharyngeal carcinoma (NPC).

**Methods:**

We studied the expression of DAB2 in NPC cell lines, xenografts and primary tumour samples. The status of promoter methylation was assessed by methylation specific PCR and bisulfite sequencing. The functional role of DAB2 in NPC was investigated by re-introducing DAB2 expression into NPC cell line C666-1.

**Results:**

Decrease or absent of *DAB2 *transcript was observed in NPC cell lines and xenografts. Loss of DAB2 protein expression was seen in 72% (33/46) of primary NPC as demonstrated by immunohistochemistry. Aberrant *DAB2 *promoter methylation was detected in 65.2% (30/46) of primary NPC samples by methylation specific PCR. Treatment of the DAB2 negative NPC cell line C666-1 with 5-aza-2'-deoxycytidine resulted in restoration of DAB2 expression in a dose-dependent manner. Overexpression of DAB2 in NPC cell line C666-1 resulted in reduced growth rate and 35% reduction in anchorage-dependent colony formation, and inhibition of serum-induced c-Fos expression compared to vector-transfected controls. Over expression of DAB2 resulted in alterations of multiple pathways as demonstrated by expression profiling and functional network analysis, which confirmed the role of DAB2 as an adaptor molecule involved in multiple receptor-mediated signalling pathways.

**Conclusions:**

We report the frequent down regulation of DAB2 in NPC and the promoter hypermethylation contributes to the loss of expression of DAB2. This is the first study demonstrating frequent DAB2 promoter hypermethylation in human cancer. Our functional studies support the putative tumour suppressor effect of DAB2 in NPC cells.

## Background

Nasopharyngeal carcinoma (NPC) poses one of the serious health problems in Southern China, including Hong Kong. It is the fifth commonest cause of cancer deaths in our male population and affects a younger age population (< 45 years old) than most of other cancers. The annual incidence rate in Hong Kong is 29.8/100,000 (Hong Kong Cancer Registry 2007; http://www3.ha.org.hk/cancereg/e_stat.asp), in great contrast to those among Caucasians in other countries (< 1/100,000) [1]. The reason of the peculiar geographic distribution remains unclear. The environmental factors and the strong association with Epstein-Barr virus (EBV) have been implicated [1]. Understanding of the molecular basis of this cancer is essential to derive effective markers for early diagnosis and targeted therapies.

Human disabled-2 (*DAB2*) encodes a 96 kDa mitogen responsive phosphoprotein that is one of the two mammalian orthologues of the drosophila disabled protein. It contains a proline-rich, SH3-binding domain (PRD) in its C-terminus, and a phosphotyrosine-binding (PTB)/-interacting domain (PID) in its N-terminus. The C-terminal PRD interacts with Grb2 by interrupting the binding of Grb2 and SOS, potentially suppressing the mitogenic signalling via Ras pathway [2,3]. It also binds clathrin, the clathrin-adaptor protein AP2 and myosin VI, facilitating clathrin-coated pit assembly and receptor-mediated endocytosis [4,5]. The endocytic and vesicular trafficking function of DAB2 are postulated to mediate its effects on cellular signalling. The conserved N-terminal PTB of DAB2 binds to members of the low-density lipoprotein receptor family [5] and transforming growth factor-β (TGF-β) type I and II receptors [6], as well as with the Ras GAP DIP1/2 [7]. The association of DAB2 with multiple signalling proteins and the lack of intrinsic catalytic enzyme activity suggest that it is an adaptor molecule involved in multiple receptor-mediated signalling pathways that plays a pivotal role in the cellular homeostasis.

DAB2 is a putative tumour suppressor and plays an important regulatory role in cellular differentiation. Induction of differentiation in the absence of DAB2 expression commits the cell to apoptosis [8]. Recently it is reported that DAB2 functions as a negative regulator of canonical Wnt signalling by stabilized beta-catenin degradation complex [9]. Decreased expression of DAB2 has been demonstrated in several cancers including ovarian, breast, prostate, oesophagus, urinary bladder, colon and choriocarcinoma [10-17]. Ectopic expression of DAB2 reduced in vitro tumour growth in ovarian, prostatic and choriocarcinoma cell lines [13,18,19] and significantly reduced the ability to form tumours in nude mice when stably expressed in ovarian cancer cells [10]. The involvement of DAB2 in nasopharyngeal carcinoma (NPC) has not been addressed before. We found that *DAB2 *transcript was absent or significantly down-regulated in NPC xenografts and cell lines comparing to immortalized normal nasopharyngeal epithelial cell lines. The protein expression in primary NPC was also significantly reduced. The differential expression patterns pointed to a possible tumour suppressor role of DAB2 in NPC. In the current study, we aimed to investigate the functional role of DAB2 in NPC carcinogenesis, and to delineate the mechanisms leading to the down-regulation of DAB2.

## Methods

### Cell lines, xenografts, and primary NPC tissues

NPC cell lines (C666-1, HK1 and HONE1), xenografts (X2117, X666, C15, C17, X1915) were maintained as described previously [20]. An SV40 large T oncogene immortalized normal nasopharyngeal epithelial cell line NP69 was also included in this study [21]. The NPC cell line C666-1 and all the NPC xenografts harbour EBV, whereas HK1, HONE1 and NP69 cells are EBV-negative. Forty-six formalin-fixed paraffin-embedded primary NPC biopsy samples were retrieved from tissue bank of the Department of Anatomical and Cellular Pathology at Prince of Wales Hospital, Hong Kong. All specimens were taken before treatment and were histologically evaluated to be EBV-positive undifferentiated carcinomas as demonstrated by EBER in situ hybridization. The male to female ratio of the patients was 3.1:1. The age of the patients ranged from 16 to 84 years with the median age of 48. Based on UICC staging classification, 5 patients had Stage I disease (11%), 14 patients had Stage II (30%), 10 patients had Stage III (22%), 17 patients had Stage IV disease (37%). The median follow-up time was 57.8 months (range 13.8-95.5 months). The study protocol was approved by the Joint CUHK-NTE Clinical Research Ethics Committee, Hong Kong.

### 5-Aza-2'-deoxycytidine (5-aza) and Trichostatin A (TSA) treatment

NPC cell line C666-1 was treated with 1, 5, 10, 15 μM of 5-Aza-2'-deoxycytidine (Sigma, St. Louis, MO) for 3 days. Culture medium with fresh 5-aza was replenished every 24 hours. For TSA treatment, cells were incubated with 50, 100 and 200 ng/ml TSA (Sigma) for 24 hours. Synergistic effect of 5-aza and TSA was tested by treating cells with 5 μM of 5-aza for 3 days followed by 100 ng/ml of TSA for 24 hours.

### Immunohistochemistry

Immunohistochemistry was performed on the Ventana Nex ES automated stainer (Ventana Corporation, Tucson, AZ) using anti-DAB2 antibody (1:50, Santa Cruz Biotechnology, Santa Cruz, CA). The cytoplasmic expression of DAB2 was assessed by assigning a proportion score and an intensity score. The proportion score was according to proportion of tumour cells with positive cytoplasmic staining (0, none; 1, < = 10%; 2, 10 to < = 25%; 3, > 25 to 50%; 4, > 50%). The intensity score was assigned for the average intensity of positive tumour cells (0, none; 1, weak; 2, intermediate; 3, strong). The cytoplasmic score of DAB2 was the product of proportion and intensity scores, ranging from 0 to 12. The cytoplasmic expression was categorized into low (score 0 to 3), intermediate (score 4-6), and high (score 7-12). The scoring was independently assessed by two pathologists (K.F.T. and A.W.C.)

### Bisulfite sequencing and methylation specific PCR (MSP)

Bisulfite sequencing and MSP has been described previously [22,23]. A total of 47 CpG sites spanning approximately 800-bp on the 5'CpG island of *DAB2 *gene were analyzed by bisulfite sequencing (Additional file 1 Figure S1). This region covered the critical transcriptional regulatory domains sufficient for DAB2 expression in epithelial cells [24,25]. The PCR Primers are listed in Table 1.

**Table 1:** PCR primers used for bisulfite sequencing and methylation specific PCR

	Primer sequence	Product size
Bisulfite sequencing		
Region1 Forward	5'-TAGTTTTTTGTTTAAAGGGTTTTAACGGGT-3'	365 bp
Region1 Reverse	5'- ACCTAAACTTAATAACTCCCCCTCA -3'	
		
Region2 Forward	5'- ATTTTGGTATATTTTTGGGGAGTTT-3'	360 bp
Region2 Reverse	5'- CCCAAACACAAAATCTCATTTCTA -3'	
		
Methylation specific PCR		
Methylated Forward	5'-ATTTTTCGTCGGGAGTGGTC-3'	79 bp
Methylated Reverse	5'-GCAACGAATACGACGAACCT-3'	
		
Unmethylated Forward	5'-GGGAGTGGTTGTGTGGTTTT-3'	103 bp
Unmethylated Reverse	5'-AACTTGGGGACACCCAAA-3'	

### Restoration of DAB2 expression in C666-1 cells

Full length *DAB2 *was amplified from TrueORF cDNA clone (Origene, Rockville, MD) and inserted into pcDNA3.1(+) (Invitrogen) to produce *DAB2 *expression vector. pcDNA3.1(+)/*DAB2 *were transfected into C666-1 cells using FuGENE HD (Roche, Mannheim, Germany). Restoration of DAB2 expression was confirmed by western blot analysis using DAB2 antibody (1:4000, Santa Cruz Biotechnology, Santa Cruz, CA, USA).

### Flow cytometric analysis

Cells transfected with pcDNA3.1(+) or pcDNA3.1(+)/*DAB2 *were subjected to flow cytometric analysis as described previously [22].

### Cell proliferation and monolayer colony formation assay

Cell proliferation of DAB2 transfected C666-1 cells was assessed using CellTiter 96^® ^Non-Radioactive Cell Proliferation Assay (MTT) (Promega, Madison, WI) according to the manufacturer's instructions. For monolayer colony formation assay, C666-1 cells were culture overnight in 60 mm plates and transfected with pcDNA3.1(+)/*DAB2 *or empty vector. Forty-eight hours later the transfectants were re-plated in triplicate and cultured for 21 days in selection medium containing G418 (500 μg/ml). Surviving colonies were stained with Gentian violet after methanol fixation and counted. The experiments were repeated in triplicate.

### Microarray analysis

Gene expression analysis of C666-1 transiently transfected with pcDNA3.1(+)/*DAB2 *for 24 hours as compared with cells transfected with empty vectors as controls were subjected to microarray analysis using 4 × 44 K Whole Human Genome Oligo Microarray system (Agilent Technologies, Santa Clara, CA). The experiment was done in duplicate. Microarray slides were scanned using Agilent G2505C microarray scanner (Agilent). The images were quantified with Feature Extraction Software (Agilent Technologies). The raw data were quantile normalized by robust multiarray average (RMA) algorithm and analyzed in Partek Genomics Suite 6.4 (Partek, St. Charles, MO). Differential gene expression was evaluated using one-way ANOVA. A fold-change cut-off of 1.5 and p < 0.01 was set to identify differentially expressed genes. The Ingenuity Pathways Analysis (IPA, Ingenuity^® ^Systems, http://www.ingenuity.com) was utilized to identify network of interacting genes and the biological functions that were most significant to the data set. Fisher's exact test was used to calculate a p-value determining the probability that each biological function assigned to that data set is due to chance alone. A p-value of less than 0.01 was considered significant.

### Quantitative real-time PCR (QRT-PCR)

QRT-PCR was performed using premixed primers and probes from TaqMan^® ^Gene Expression Assays (Appliedbiosystems, Foster city, CA) and run on ABI 7500 real time fast PCR system (Appliedbiosystems). All reactions were done in triplicate. The relative expression level was normalized with beta-actin and calculated using the 2^(-Delta Delta Ct) method [26].

### Western blot analysis

MAP kinase pathways were studied by western blotting according to standard procedures as described previously [22,23]. Anti-ERK1/2, anti-phospho-ERK1/2 and anti-c-Fos antibodies came from Cell Signalling Technology, Inc. (Beverly, MA). Anti-GAPDH (Calbiochem, La Jolla, CA) was used as a loading control.

### Statistical Analysis

Mann-Whitney U test was used to compare the data and p value < 0.05 was considered as statistically significant. Statistical analyses were performed using SPSS 16.0 software (SPSS Inc., Chicago, IL, USA).

## Results

### Loss of DAB2 expression in NPC

**Figure 1:**
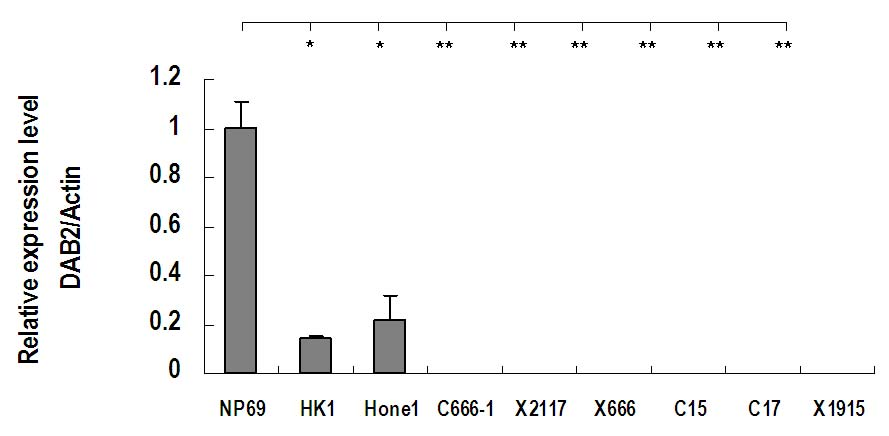
***DAB2 *mRNA expression in NPC cell lines, xenografts and normal nasopharyngeal epithelial cell line**. The QRT PCR was done in triplicate. The error bars represent the standard deviations. P value (* p < 0.05, **p < 0.001) refers to the comparison with normal nasopharyngeal epithelial cell line NP69.

We used QRT-PCR assay to assess the *DAB2 *mRNA expression level in NPC cell lines, xenografts and immortalized normal nasopharyngeal epithelial cell line. As shown in Figure 1, *DAB2 *expression was undetectable in NPC cell line C666-1 and all 5 NPC xenografts (X2117, X666, C15, C17 and X1915). In NPC cell lines HK1 and HONE1, the *DAB2 *expression levels were significantly reduced comparing with the expression level of immortalized normal nasopharyngeal epithelial cell line.

**Figure 2:**
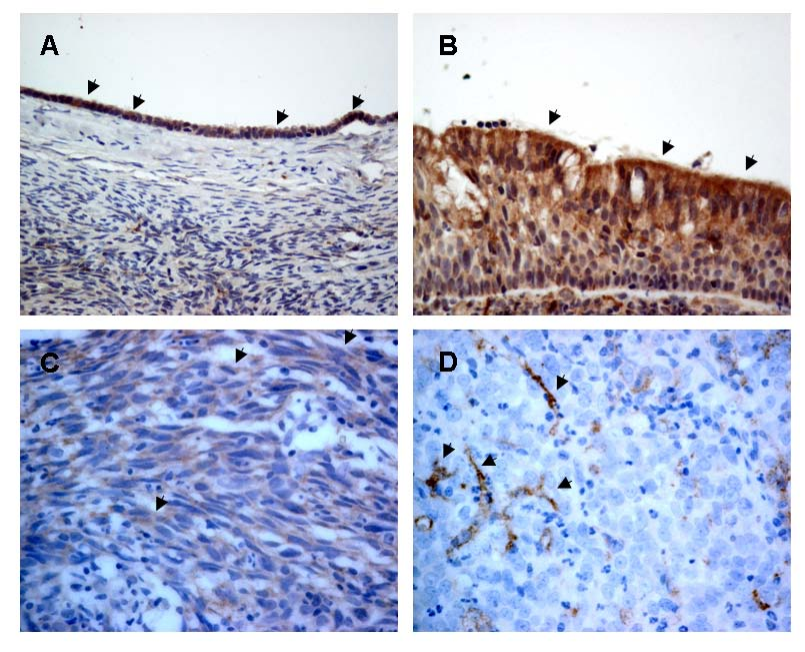
**Immunohistochemical analysis of DAB2 expression in NPC**. (A) Surface epithelium of ovary served as positive control (original magnification × 400). (B) Normal nasopharyngeal epithelium (× 400). Arrows indicate the positive epithelium. (C) Cytoplasmic staining in a NPC biopsy (× 200). Arrows indicate positive NPC cells. (D) Negative staining in NPC cells. The dendritic cells (arrows) in the stroma served as internal positive controls (× 200).

Immunohistochemistry was performed in 46 NPC biopsy samples to determine the DAB2 protein expression in primary NPC. We first optimized the staining condition using human ovarian tissue. Intense DAB2 immunoreactivity was observed in the ovarian surface epithelial cells (Figure 2A). In normal nasopharyngeal epithelium, intermediate to high level of cytoplasmic stain was observed as demonstrated in Figure 2B. In NPC biopsy samples, thirty-three cases (72%, 33 out of 46) were negative for DAB2 stain. Focal immunoreactivity was observed in the remaining 13 NPC biopsies and all were scored as low level expression (score <4). Examples of positive and negative DAB2 immunoreactivity in NPC biopsies were shown in Figurea 2C and 2D, respectively. The dendritic cells distributed in the stroma showed strong immunoreactivity for DAB2. It has been reported that macrophage and fibroblast-like cells in the stroma expressed DAB2 [12,27]. Therefore, they served as internal positive controls for the staining as shown in Figure 2D. DAB2 immunoreactivity was not associated with age, gender, stage of disease, disease specific survival or overall survival of the patients.

### Promoter methylation contributes to *DAB2 *down regulation in NPC

**Figure 3:**
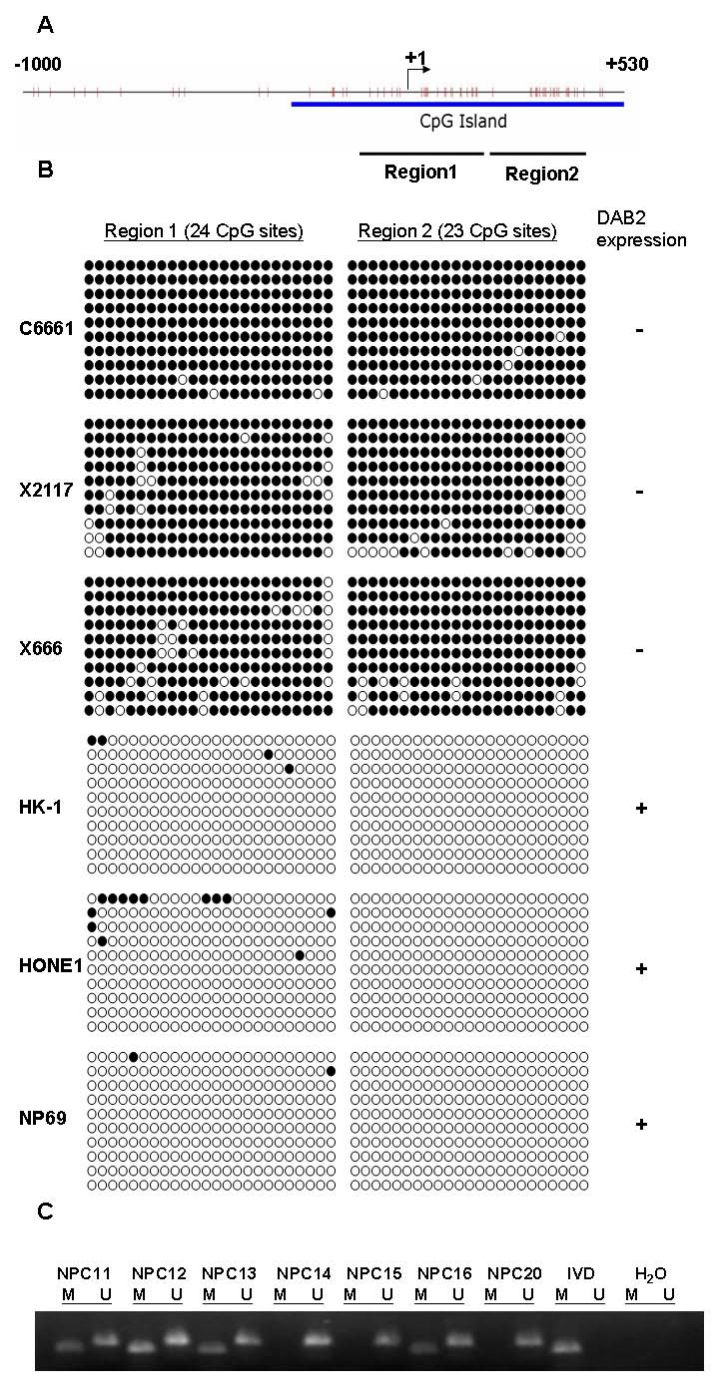
**Promoter methylation of *DAB2 *in NPC**. (A) The CpG island of *DAB2*. (B) Bisulfite sequencing of *DAB2 *CpG island. Open circles represent unmethylated CpG sites; filled circles represent methylated CpG sites. (C) Representative MSP results in primary NPC samples. M: methylated allele; U: unmethylated allele. IVD: In vitro methylated DNA.

Sheng et al. characterized human *DAB2 *gene and identified a CpG island (39 CpG sites) spanning 420-bp sequence 5' of exon 1. They found that this 420-bp putative promoter contains the transcription start site and is sufficient for active transcription in epithelial cells [24]. Since the expression of *DAB2 *was down-regulated in NPC, we examined whether the gene was silenced by promoter hypermethylation. Bisulfite sequencing was performed to determine the methylation pattern of the 5'CpG island of *DAB2 *in NPC cell lines and xenografts. Two regions were amplified for analysis: region 1 with 24 CpG sites and region 2 with 23 CpG sites (Figure 3A and Additional file 1 Figure S1). As shown in Figure 3B, dense methylation in both region 1 and region 2 was observed in NPC cell line C666-1, NPC xenografts X2117 and X666, in which no *DAB2 *mRNA expression was detected. In NPC cell lines HK1 and HONE1, and immortalized normal nasopharyngeal epithelial cell line NP69, where the *DAB2 *expression was retained, the promoter region was unmethylated. These results indicated that aberrant methylation contributed to the loss of *DAB2 *expression in NPC cell lines and xenografts.

We further analyzed the *DAB2 *promoter methylation status in primary NPC tissues by MSP. Aberrant methylation on the promoter region of *DAB2 *was detected in 65.2% (30/46) of NPC primary tumour biopsy samples. Representative results were shown in Figure 3C. Our data suggested that aberrant promoter methylation is a common event that contributes to the down regulation of *DAB2 *expression in NPC. However, no correlation could be demonstrated between *DAB2 *hypermethylation and clinicopathologic features including age, gender, stage of disease, disease specific survival or overall survival of the patients.

**Figure 4:**
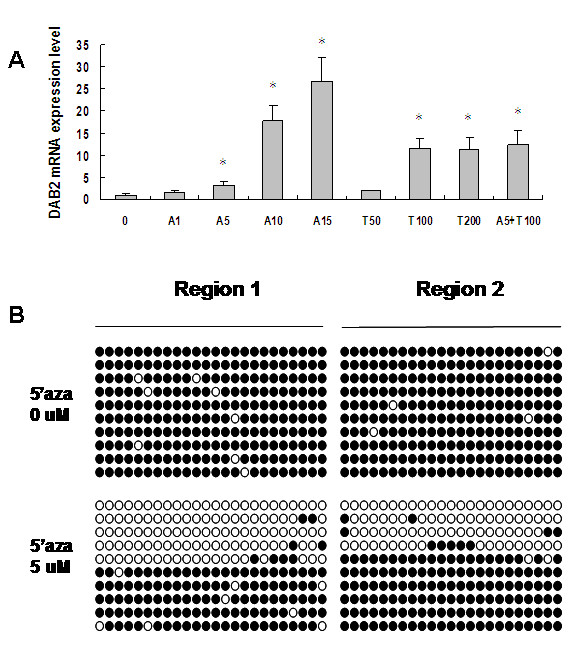
**Restoration of DAB2 expression by 5-aza and TSA in C666-1 cells**. (A) The relative *DAB2 *mRNA expression was calculated comparing each sample to no treatment controls. * p < 0.05. A1 - A15, T50 - T200 and A5+T100 refer to the concentrations of A (5-aza) and T (TSA). The treatment protocol has been described in method section. (B) Bisulfite sequencing of *DAB2 *promoter on C666-1 cells before and after 5-aza treatment.

We treated NPC cell line C666-1, which shown complete loss of *DAB2 *expression, with demethylation agent 5-aza-2'-deoxycytidine. *DAB2 *mRNA expression was restored in a dose dependent manner as demonstrated by quantitative real time PCR (Figure 4A). Using bisulfite sequencing, partial demethylation of *DAB2 *promoter was detected in C666-1 cells after 5-aza treatment (Figure 4B). The promoter region we analyzed included the major GATA6 *cis *elements that are critical for the transcriptional regulatory of *DAB2 *gene [25]. The finding further confirmed that *DAB2 *down regulation was mediated by promoter methylation. Induction of *DAB2 *transcription by histone deacetylase inhibitor has been reported previously [19]. In C666-1 cells, histone deacetylases inhibitor (HDACi) trichostatin A (TSA) could also restore the *DAB2 *mRNA expression (Figure 4A). However, we didn't observe a synergistic induction of *DAB2 *transcript after combined treatment with 5-aza and TSA in comparison to TSA treatment alone (Figure 4A).

### DAB2 re-expression reduced *in vitro *growth of C666-1 cells

**Figure 5:**
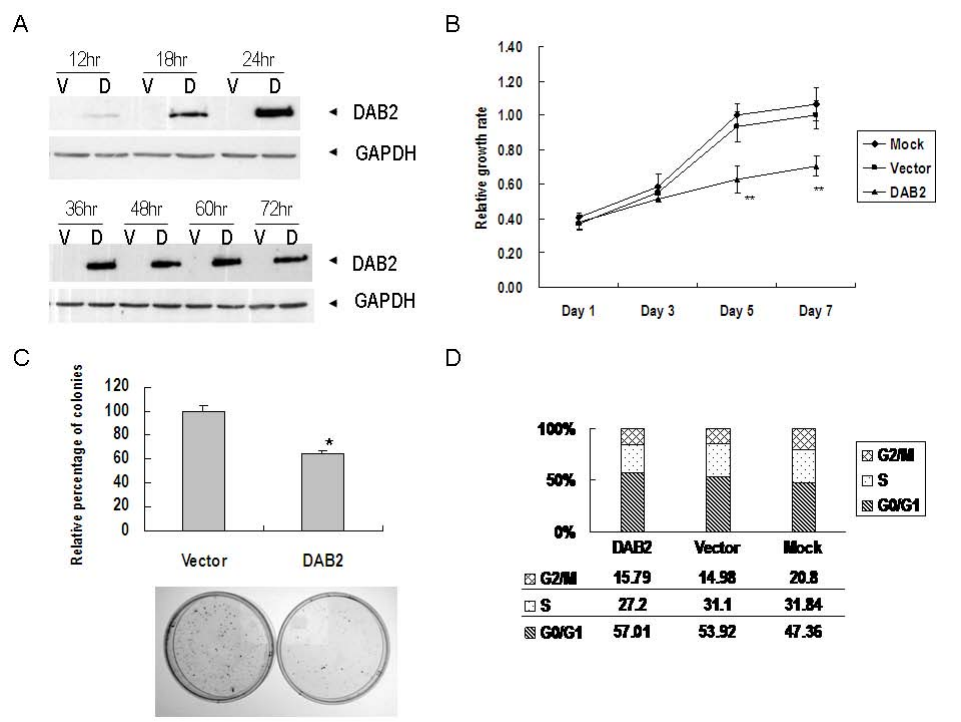
**Re-expresssion of DAB2 reduced *in vitro *growth of C666-1 cells**. (A) Western blot analysis of DAB2 transfected C666-1 cells. V: vector control; D: DAB2 transfectant. (B) Cell proliferation as determined by MTT assay. The mean and SD obtained from five experiments were plotted. ** (p < 0.01). (C) Anchorage-dependent colony formation assay. The experiment was done in triplicate and the error bars represent standard deviations. *p < 0.05. (D) Cell cycle distribution of C666-1 cells transfected with DAB2, vector control and mock transfected cells by flow cytometry analysis.

To evaluate the potential tumour suppressor effect of DAB2 in NPC, we transfected full length *DAB2 *cDNA in pcDNA3.1(+) expression vector into C666-1 cells. The expression of transfected cDNA was confirmed by western blot analysis. The endogenous DAB2 expression was completely loss in wild type C666-1 cells. Re-expression of DAB2 could be detected at 12 hours after transfection and remained at high level after 72 hours (Figure 5A). MTT assay was done to assess the proliferation rate of the transfectants (Figure 5B). DAB2 suppressed the proliferation of C666-1 cells in comparison to vector controls and mock transfection controls (transfection reagent only). We further evaluated the growth inhibitory effect of DAB2 in NPC cells using anchorage-dependent colony formation assay. C666-1 cells transfected with pcDNA3.1(+)/*DAB2 *or empty vector were selected with G418 for 21 days. Exogenous DAB2 resulted in 35% reduction in colony formation compared to vector-transfected control (Figure 5C). Since a growth inhibitory effect was observed in DAB2 transfected NPC cells, we analyzed the transfectants for cell cycle parameters using flow cytometry. Twenty-four hours after transfected with pcDNA3.1(+)/*DAB2*, empty vector, or mock transfection, the C666-1 cells were harvested and analyzed. Under our experimental condition, DAB2 did not cause significant change in cell cycle parameters, although a slightly reduction (3.9%) of S phase cells was observed in DAB2 expressing cells (Figure 5D).

### DAB2 inhibited serum-induced c-Fos expression

**Figure 6:**
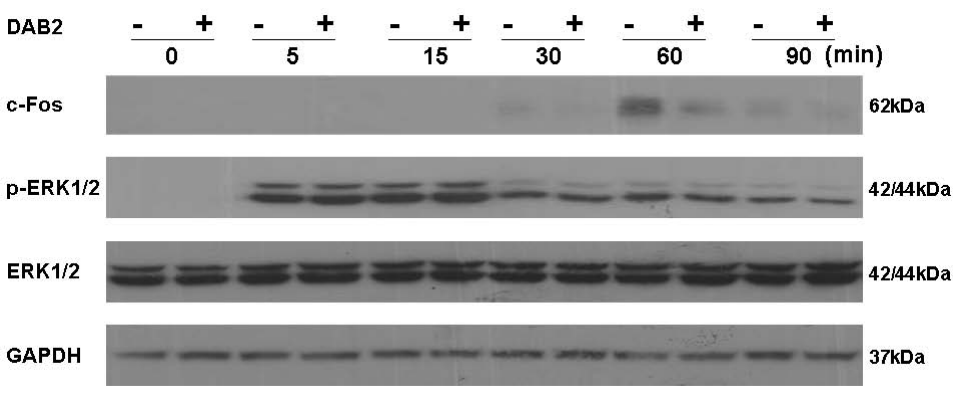
**Transfection of DAB2 inhibited serum-induced c-Fos expression**. Twenty-four hours after transfection with vector or DAB2, C666-1 cells were cultured without serum for 18 hours and then stimulated with serum for 0, 5, 15, 30, 60, 90 minutes. Cells were immediately washed with cold phosphate-buffered saline and collected by scraping. The cell lysates were analyzed for MAP kinase pathway by western blotting using anti- total and phospho-ERK1/2 and c-Fos.

DAB2 was identified as a mitogen responsive phosphoprotein and its growth suppression function is thought to be secondary to the inhibition of MAP kinase pathway [28]. To explore the mechanism for tumour suppression by DAB2 in NPC, we studied the effect of DAB2 on serum-induced MAP kinase signalling in NPC cells C666-1. We transfected C666-1 cells with empty vector or DAB2 expression vector. The cells were allowed to recover for 24 hours, followed by starvation in serum-free medium for another 18 hours, and then stimulated with 20% serum in culture medium. The cells were harvested at various time points and assayed for MAP kinase pathway by western blotting. C666-1 cells exhibited no basal MAP kinase activity after starvation (Figure 6). Activation of MAP kinase, as demonstrated by phospho-ERK1/2 immunoblot, was observed at 5 minutes after serum stimulation and attenuated after 30 minutes. MAP kinase activation was not affected by DAB2 expression. The p-ERK1/2 activities were unchanged upon serum stimulation in transient DAB2-transfected cells compared with vector-transfected cells. C-Fos was induced at 30 minutes after serum stimulation and reached the maximal level at 60 minutes. In DAB2 expressing C666-1 cells, a much weaker c-Fos induction was observed compared with vector-transfected control cells. C-Fos is an immediate early gene that is required for malignant tumour growth. DAB2 might exert the tumour suppression function by inhibiting c-Fos expression in NPC.

### Identification of cellular networks and pathways regulated by DAB2

To search for potential genes and pathways regulated by DAB2 in NPC, we compared the expression profile of C666-1 cells overexpressing DAB2 to vector control-transfected cells. The expression of DAB2 protein after transfection was confirmed by western blotting. The expression profile was performed using Agilent 4 × 44K whole human genome oligonucleotide (60 mer) array. Differential gene expression was evaluated using one-way ANOVA. Within the ANOVA model, linear contrasts were used to compare the transiently transfected cells carrying DAB2 to the vector control. To adjust for multiple testing errors, a step-up Benjamini false discovery rates (FDR) adjustment was used. FDR was set to be less than 20% to produce a list of significantly differentially expressed genes for each contrast described above.

A total of 628 differentially expressed genes were identified at expression cutoff of 1.5 fold and p = 0.01. Top 50 significantly up-regulated and down-regulated genes were listed in Additional file 2 Table S1. Some of the genes were confirmed by real time QRT-PCR (Additional file 2 Table S2). Each gene identifier was mapped to its corresponding gene object in the Ingenuity Pathways Knowledge Base. Two hundred and nine of these genes, called focus genes, were overlaid onto a global molecular network developed from information contained in the Ingenuity Pathways Knowledge Base. Networks of these focus genes were then algorithmically generated based on their connectivity. A total of 32 partially overlapped networks were identified. Of which 9 networks involved more than 1 focus gene. Top functions of these genes were related to cancer, cell death, endocrine systems disorders, cellular and organismal development, cell-to-cell signalling and interaction, grow and proliferation, cell cycle and cell morphology. Additional file 1 Figure S2 shows the most significant three gene networks. The first Network, which was built from 22 genes and received the highest IPA score, was centred on MAPK, RAS, AKT and IGF1 and associated with cancer, cell death and endocrine system disorders. The second network, incorporating 22 genes, was centred on NFκB and AP1, and associated with endocrine system disorders, metabolic diseases. The third network built on 23 genes was mostly centred around ERK and associated with cardiovascular, cellular and organismal development.

The use of IPA identified the mitotic roles of Polo-like kinase, Wnt/beta-cantenin signalling and cell cycle regulation by BTG family proteins as the most significant canonical pathways altered upon DAB2 transfection (Table 2, Additional file 1 Figure S3). All three canonical pathways were down-regulated in DAB2-expressing C666-1 cells.

**Table 2:** Top three canonical pathways in DAB2-expressing C666-1 cells

Name	p-value	Ratio	Molecules
Mitotic roles of Polo-like Kinases	1.96E-03	13/62 (0.21)	ANAPC2, ANAPC5, ANAPC13 CCNB1, CDC25A, HSP90AB1, PPP2R4, PPP2R1B, PPP2R2A, PPP2R2C, PPP2R5E, SMC1A, TGFB1
Wnt/beta-catenin Signalling	8.83E-03	26/165 (0.158)	AKT1, APC2, DKK2, DKK3, DVL1, FZD9, FZD10, GNAQ, HDAC1, HNF1A, MDM2, PPP2R4, PPP2R1B, PPP2R2A, PPP2R2C, PPP2R5E, SOX8, SOX5, TGFB1, UBB, UBD, WIF1, WNT2, WNT16, WNT5B, WNT8B
Cell cycle regulation by BTG Family Proteins	1.78E-02	8/36 (0.222)	CCNE1, CDK4, E2F6, PPP2R4, PPP2R1B, PPP2R2A, PPP2R2C, PPP2R5E

## Discussion

Although decrease or absent of DAB2 has been reported in several cancer types, the status in NPC has not been explored. In this study, we screened NPC cell lines and xenografts for DAB2 expression level and observed a significant down-regulation of *DAB2 *transcript level in NPC cells comparing to immortalized normal nasopharyngeal epithelial cells. We further examined the DAB2 protein expression level in primary NPC biopsy samples using immunohistochemistry. DAB2 expression was retained in normal nasopharyngeal epithelium. However, complete loss of DAB2 expression was seen in 72% of primary NPC. The frequent down-regulation of DAB2 in NPC implied that it might be a tumour suppressor gene involved in NPC pathogenesis.

Several genetic and epigenetic processes contribute to the silencing of tumour suppressor genes, including loss of genetic materials, mutation, promoter methylation, histone acetylation and post-transcriptional regulation by micro RNAs, etc. Loss of heterozygosity and deletion of chromosome 5p13, where *DAB2 *is located, is uncommon in NPC according to our previous studies [29-31]. Since *DAB2 *down-regulation was observed in transcription level, post-transcriptional regulation by micro RNAs is unlikely. In colorectal and gastric cancer cell lines, GATA4 and GATA5 genes and their target genes including *DAB2 *promoter were found to be hypermethylated, suggesting epigenetic silencing may be responsible for loss of DAB2 protein expression in these cell lines [32]. Zhou et al reported that the transcription regulation of *DAB2 *is mediated through histone acetylation and by specific transcription factors such as GATA6 but not by DNA methylation in urothelial carcinoma [16,19]. They found a critical regulatory region on *DAB2 *promoter (-295 and -120-bp upstream from exon 1) that contains the major GATA6 *cis *elements. We sequenced the promoter region of *DAB2 *gene spanning the critical regulatory domains described by Zhou et al and no mutation was found in NPC cell lines and xenografts [Genbank: HM135031 to HM135036], suggesting mechanisms other than mutation on transcription factor binding sites should contribute to the down-regulation of *DAB2*.

Previous studies suggested that DNA methylation in the promoter region of *DAB2 *is infrequent in esophageal and breast cancers [11,15]. In contrast to these studies, we found that promoter methylation of *DAB2 *is observed in 65.2% (30/46) of primary NPC. Methylation status on the promoter region was associated with the expression level of *DAB2 *in NPC cell lines and xenografts. Treatment with demethylation agent 5-aza-2'-deoxycytidine restored the *DAB2 *expression in NPC cell line in a dose-dependent manner. The results strongly suggested that promoter methylation is one of the frequent epigenetic mechanisms contributed to the inactivation of *DAB2 *in NPC.

One of the unique features of NPC in our locality is that the majority of NPC is undifferentiated carcinoma and consistently associated with Epstein Barr virus (EBV). In our previous studies, we have demonstrated the frequent hypermethylation of multiple genes in NPC including RASSF1A, RARβ, DAP-kinase, p15, p16, TSLC1, DLEC1, *etc*. [33-36]. The alterations are accompanied by methylation of EBV genome, suggesting a process of virus-associated hypermethylation in cancer development. EBV-associated gastric cancer showed global CpG island methylation on promoter region of various cancer-related gene and demonstrated methylation phenotype [37]. The mechanisms by which EBV infection promotes DNA methylation are largely unknown. Recent studies suggested that EBV latent membrane protein 1 (LMP1) activates cellular DNA methyltransferases, and therefore down-regulates the expression of critical host genes using cellular DNA methylation machinery [38].

The high frequency of epigenetic inactivation of *DAB2 *in NPC has led to the hypothesis that *DAB2 *might be a tumour suppressor gene in this cancer. In keeping with this hypothesis, we demonstrated that exogenous expression of DAB2 suppressed NPC tumour growth in vitro as demonstrated by cell proliferation assay, and reduced the anchorage-dependent colony formation. Previously, DAB2 has been found to exert the tumour suppressor effect by uncoupling MAP kinase activation and c-Fos expression [28]. In keeping with this finding, our results showed that serum-induced c-Fos expression was greatly reduced in DAB2-expressing NPC cells compared to vector-transfected controls, whereas the activity of MAP kinase did not changed. DAB2 might restrict the nuclear entry of MAP kinase by mediating the trafficking of cargos containing importins away from nuclear pore, thus preventing the activation of c-Fos by MAP kinase in nucleus [39].

To further explore the functional role of DAB2 in NPC carcinogenesis, we compared the expression profile of DAB2 expressing-C666-1 cells and the vector control-transfectants. Functional network analysis of differentially expressed genes revealed that the networks involved were centred on signalling proteins NFκB, AKT, TGFbeta, and ERK, which confirmed the role of DAB2 as an adaptor molecule involved in multiple receptor-mediated signalling pathways.

Recent studies demonstrated that DAB2 interacts with Axin and contributes to the maintenance of the differentiated state and restrain Wnt-mediated proliferation [40]. Over expression of DAB2 inhibits Wnt-3A-induced accumulation of beta-catenin, decreased Dishevelled-3 (Dvl-3)/Axin interactions and maintains Axin/beta-catenin/GSK3 interactions, and attenuates Wnt/beta-catenin-mediated signalling. In this study, pathways analysis of the microarray data confirmed the involvement of DAB2 in Wnt/beta-catenin signalling pathway. Attenuation of canonical Wnt/beta-catenin signalling was observed in C666-1 cells expressing DAB2 (Additional file 1 Figure S2). Although the role of the Wnt pathway in NPC has not been fully explored, there is abundant evidence that aberrant Wnt signalling plays a role in NPC development [41]. Delineation of the regulatory role of DAB2 might provide insight into the molecular mechanisms of NPC development.

The mitotic role of Polo-like kinase is the top-scored canonical pathway identified in DAB2 expressing C666-1 cells. Polo-like kinases play critical roles during multiple stages of cell cycle progression, starting from control of the G2/M transition through phosphorylation of CDC25C and mitotic cyclins and a role in the DNA damage checkpoint adaptation to prevent entry into mitosis [42]. DAB2 is phosphorylated by CDC2 during mitosis phase, which promotes the binding of DAB2 to the peptidylprolyl isomerase Pin1 [43]. The interaction between DAB2 and Pin1 facilitates DAB2 dephosphorylation. However, little is known about the role of DAB2 in mitotic control. The observation that the mitotic role of Polo-like kinase pathway is down-regulated in DAB2 expressing C666-1 cells is of interest. DAB2 might exert its growth inhibitory effect through the regulation of mitosis. The exact role of DAB2 in mitotic control remains to be elucidated.

## Conclusion

DAB2 is a multi-function, multi-potency adaptor protein. Eliminating DAB2 expression may contribute to disruption of various signalling pathway in NPC. Further studies are needed to delineate the mechanisms underlying its tumour suppressor effect in NPC.

## Competing interests

The authors declare that they have no competing interests.

## Authors' contributions

DN, LC, KS carried out the molecular studies. PL performed the immunohistochemistry. RL was responsible for the quality control of data and algorithms. TL, MC and AC carried out the microarray analysis and statistical analysis. JT conceived of the study, and participated in its design and draft the manuscript. KL and KT participated in the design of the study, data analysis and interpretation, and manuscript review. All authors read and approved the final manuscript.

## Pre-publication history

The pre-publication history for this paper can be accessed here:

http://www.biomedcentral.com/1471-2407/10/253/prepub

## Supplementary Material

Additional file 1 **Supplementary Figures**. Figure S1 - The promoter sequence of DAB2 gene. Figure S2 - Top-scoring three networks in DAB2 overexpressing C666-1 cells. Figure S3 - Top-scoring three canonical pathways in DAB2 overexpressing C666-1 cells. Click here for file

Additional file 2 **Supplementary Tables**. Table S1 - Top 50 significantly up- and down-regulated genes in DAB2 expressing C666-1 cells. Table S2 - Expression changes in C666-1 cells transfected with DAB2. Click here for file
