# Supplementary material for: Putative tumour-suppressor gene DAB2 is frequently down regulated by promoter hypermethylation in nasopharyngeal carcinoma
Source: BMC Cancer. 2010 Jun 3;10:253. doi: 10.1186/1471-2407-10-253 (PMC2891638; doi:10.1186/1471-2407-10-253)

### Figure S1 - The promoter sequence of *DOC2/hDAB2* gene

The promoter region of *DOC2/hDAB2* contains a CpG island. The methylation pattern was investigated by bisulfite sequencing analysis. Two regions spanning ~800-bp were amplified and sequenced: region 1 with 24 CpG sites and region 2 with 23 CpG sites. The dotted lines indicate the PCR primer sites for bisulfite sequencing. The primer sequences are showed in Table 1. Exon 1 is capitalized. CpG sites are underlined and numbered. Potential GATA6 *cis* elements are marked in boldface type and the region containing critical regulatory domains [Zhou J, et al., 2005] is highlighted in yellow.

cagtcctttgttcaaagggccccaacgggttggttaggatgactccgtgtgccacat

1 2

cactcgcagtggcaatccagggaacggatctgtgaaacgaagctcggtggatcccac

3 4 5 6

ccctttcctccagagcttccctttcctGTCCTTATTTGCACCCGCCCCCCGCCCGCG

7 8 9 10

TCGGTCTGGGGCTTTTGACTTCTCCC**CGAACA**CATTTCCCCTCCCGTCGCTGGGTCC

11 12 13 14

CTG**CGATCG**CCCCCAGCTGGTGGGGCTCGCGGAGCTCAGGGGAGCGGGGTCCTCTGC

15 16 17 18 19 20

GCCGCTGCAGCGCG**AGTTAATAAACAGTTAAGTTTG**GAAGACTCTGCAGACACGTTG

21 22 23 24

AGGGGGAG**TTACCA**AGCCCAGGCAGCAAAAACATCCTGGCACATTCCTGGGGAGTCC

TCAGCTGCCAGCATC**TGATTA**GAACC**ATATCT**CTCGCCGGGAGTGGCCGCGCGGCTC

25 26 27 28 29

CGAAGCTCCCGGCCGGCGGCTATTTAAGCGAGGCCCGCCGCATCCGCTGCGCTGTAG

30 31 32 33 34 35 36 37 38

CCTGGAGGCTCCGGGCGCGGGGAAGTCATGCTCGCTTCACGGAGGCAATAGCTAGCC

39 40 41 42 43 44

Ggtgagtagcttcgaaacccttcttccctctggctccattttttttttaaagatctc

45

cttctctcctttgctccccctctctcacccccccgccccccgctacccttctttgag

46 47

tacagaccttgggctgtggggaatcttagaaatgagaccctgtgcttggg

### Figure S2 - Top-scoring three networks in DOC2/hDAB2 overexpressing C666-1 cells

Nodes represent genes, with their shape representing the functional class of the gene product, and edges indicate the biological relationship between the nodes (see legend). Nodes are color coded (red, overexpression; green, underexpression). The meanings of node shape are indicated. Solid lines imply direct relationships between proteins; dotted lines imply indirect interactions.


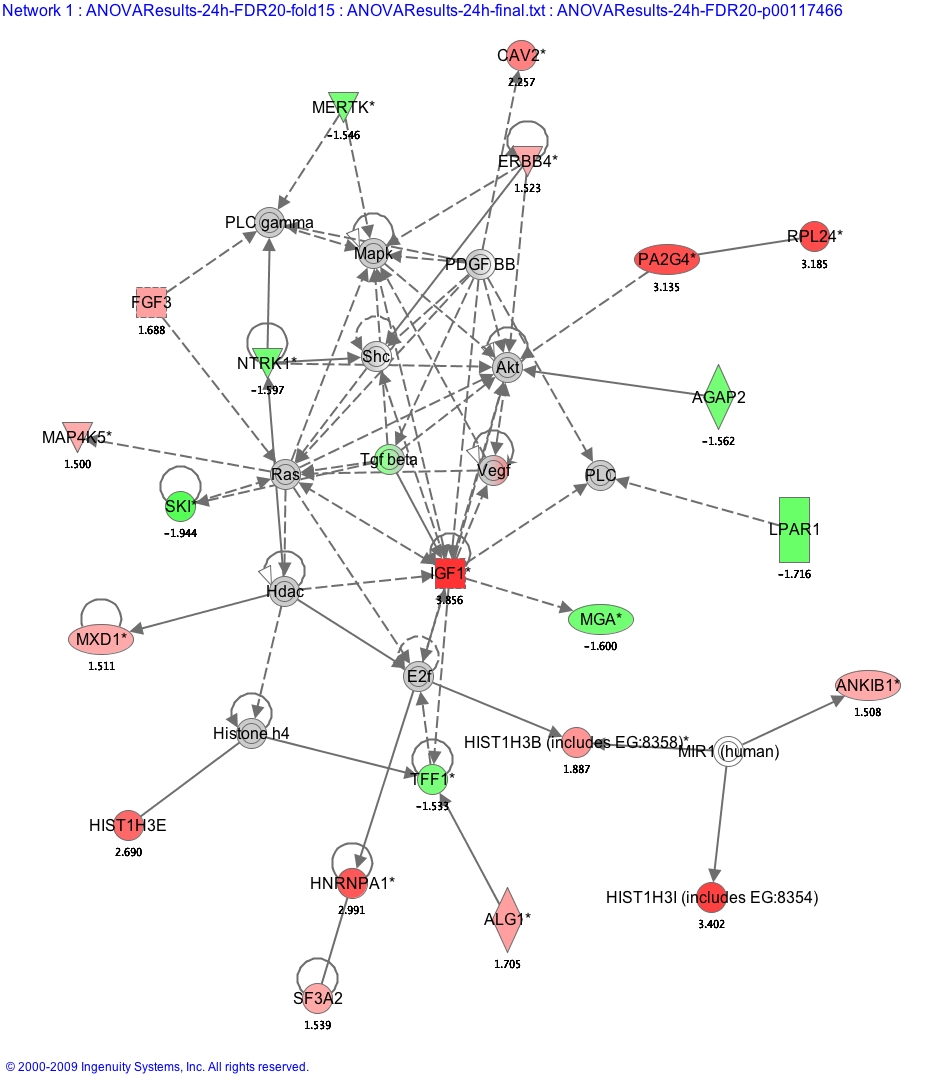

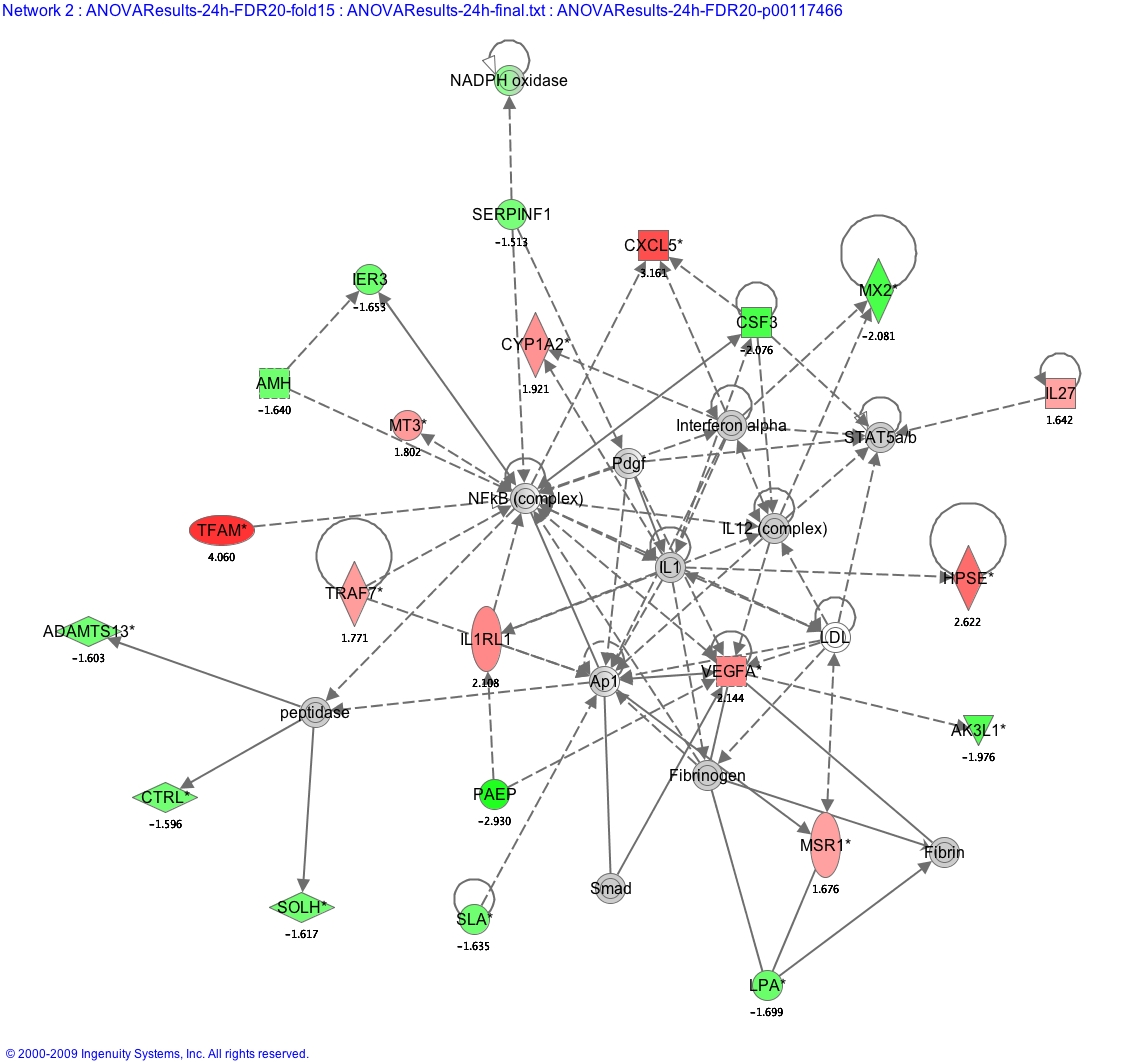

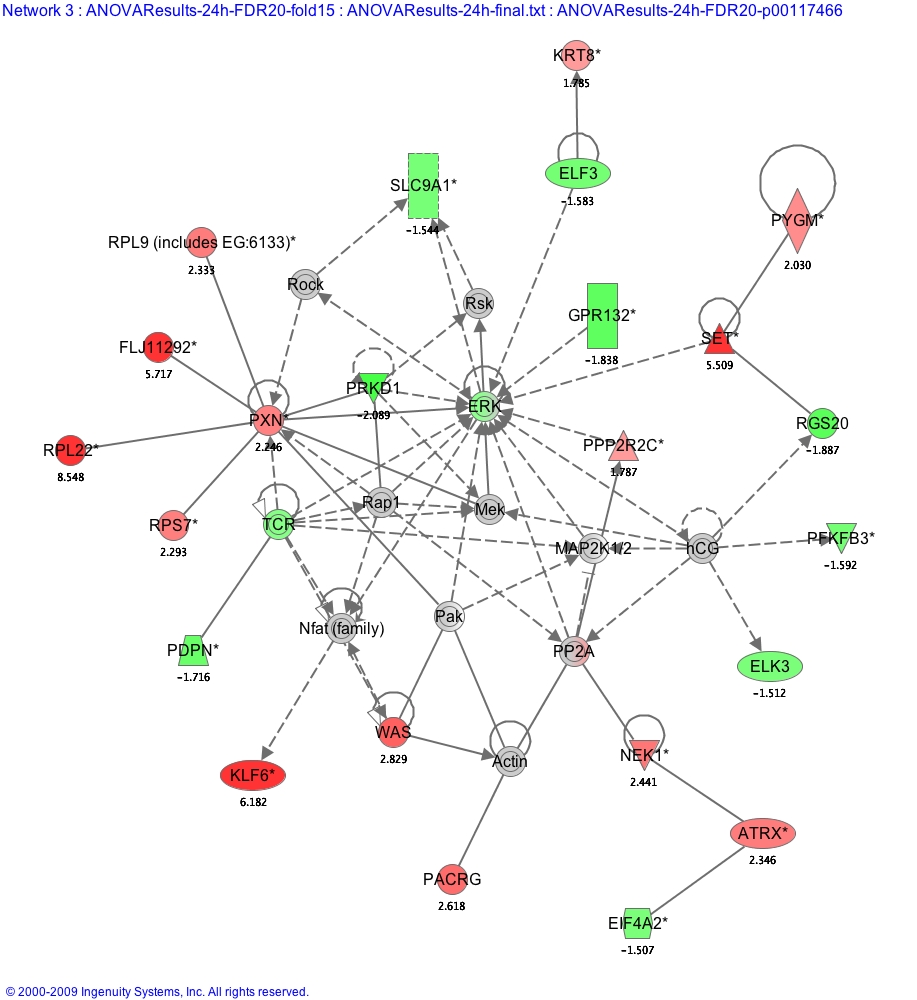


### Figure S3 - Top-scoring three canonical pathways in DOC2/hDAB2 overexpressing C666-1 cells.


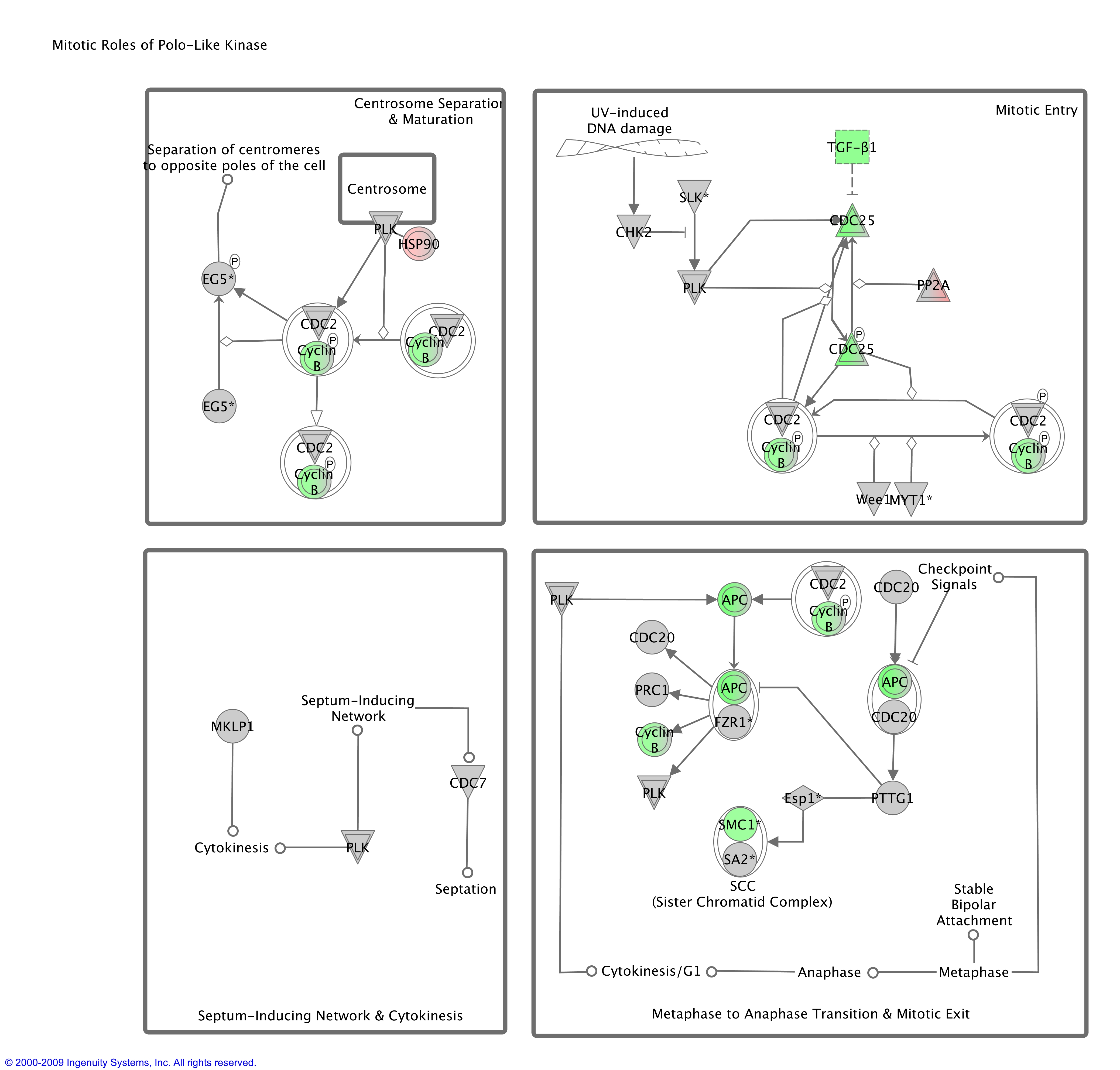

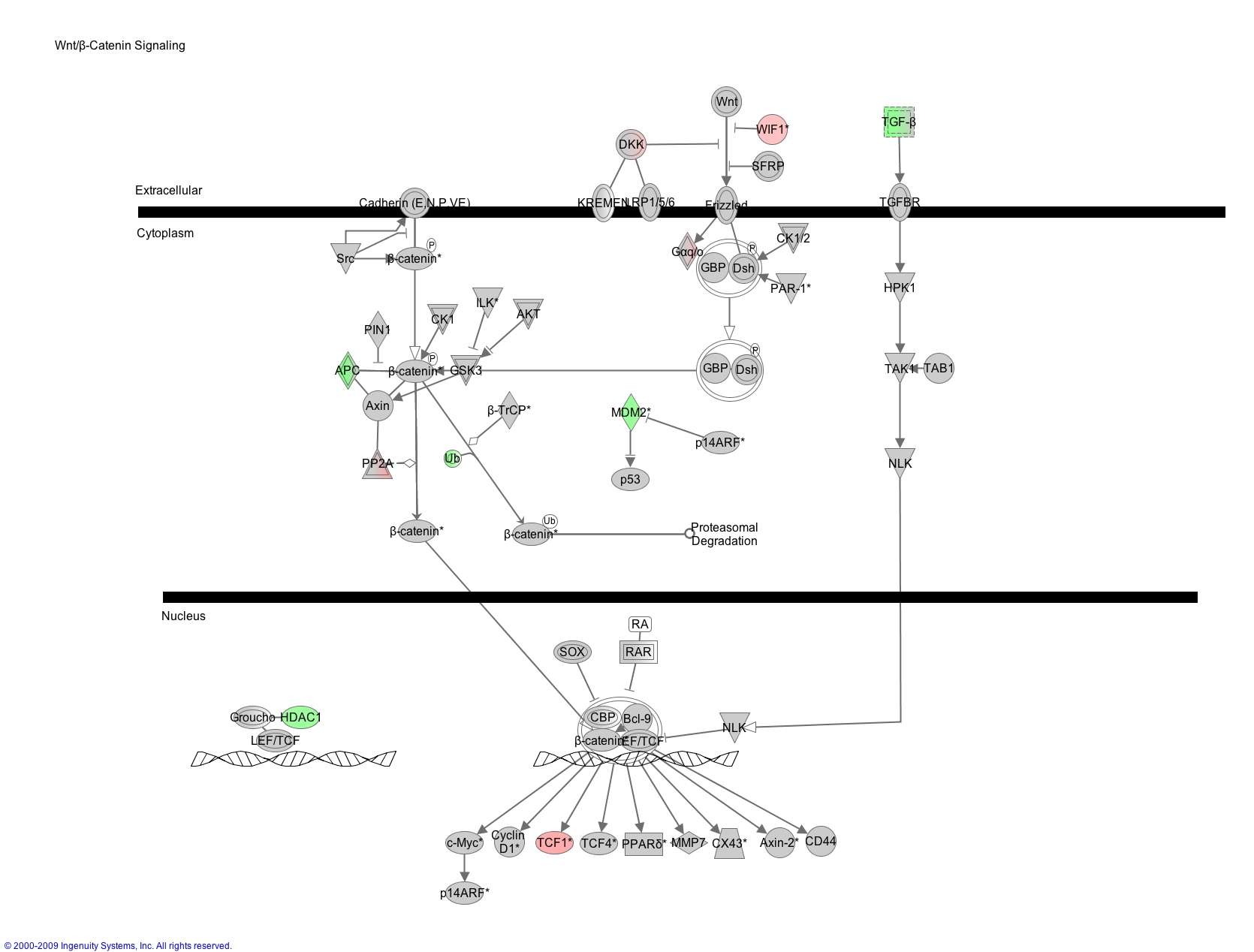

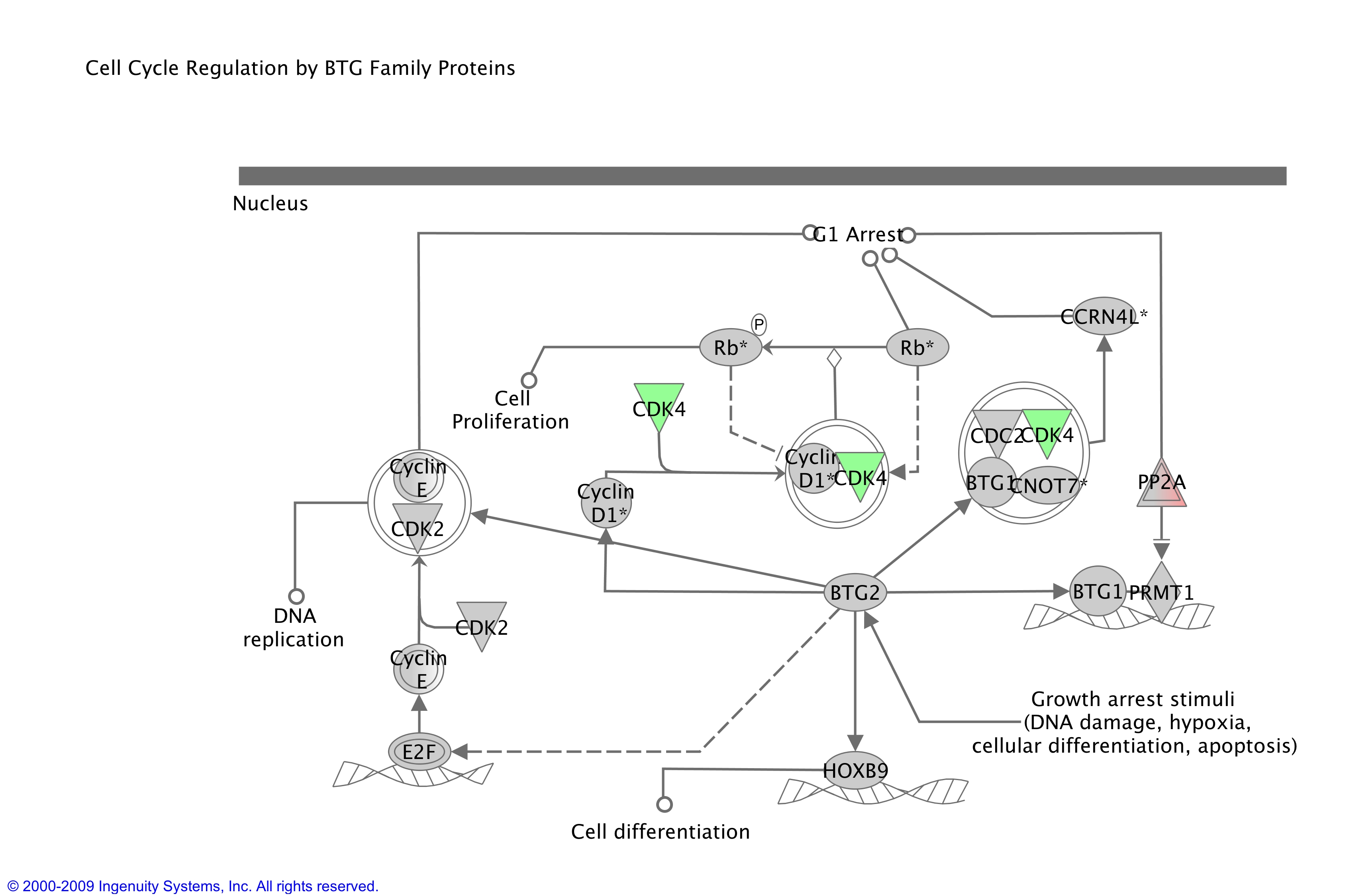

Supplement: Additional file 1 — Supplementary Figures. Figure S1 - The promoter sequence of DAB2 gene. Figure S2 - Top-scoring three networks in DAB2 overexpressing C666-1 cells. Figure S3 - Top-scoring three canonical pathways in DAB2 overexpressing C666-1 cells. [file 1471-2407-10-253-S1.DOC]
