# Supplementary material for: Putative tumour-suppressor gene DAB2 is frequently down regulated by promoter hypermethylation in nasopharyngeal carcinoma
Source: BMC Cancer. 2010 Jun 3;10:253. doi: 10.1186/1471-2407-10-253 (PMC2891638; doi:10.1186/1471-2407-10-253)
Supplement: Additional file 2 — Supplementary Tables. Table S1 - Top 50 significantly up- and down-regulated genes in DAB2 expressing C666-1 cells. Table S2 - Expression changes in C666-1 cells transfected with DAB2. [file 1471-2407-10-253-S2.DOC]

Additional file 2 Table S1 Top 50 significantly up- and down-regulated genes in DOC2/hDAB2 expressing-C666-1 cells (Fold change cutoff=1.5 , FDR<0.2).

| Down-regulated in DOC2/hDAB2 expressing C666-1 cells | |  |  |  |
| --- | --- | --- | --- | --- |
|  |  |  |  |  |
| Symbol | Entrez Gene Name | Location | Type | Fold Change |
| APOL6 | apolipoprotein L, 6 | Extracellular Space | transporter | -3.563 |
| PAEP | progestagen-associated endometrial protein | Extracellular Space | other | -2.930 |
| RBP7 | retinol binding protein 7, cellular | Cytoplasm | other | -2.750 |
| AOC2 | amine oxidase, copper containing 2 (retina-specific) | Unknown | enzyme | -2.555 |
| ZSCAN1 | zinc finger and SCAN domain containing 1 | Nucleus | other | -2.545 |
| AKAP3 | A kinase (PRKA) anchor protein 3 | Cytoplasm | other | -2.509 |
| ELSPBP1 | epididymal sperm binding protein 1 | Unknown | other | -2.403 |
| ATP2B3 | ATPase, Ca++ transporting, plasma membrane 3 | Plasma Membrane | transporter | -2.164 |
| CLEC4E | C-type lectin domain family 4, member E | Unknown | other | -2.148 |
| MYEF2 | myelin expression factor 2 | Nucleus | transcription regulator | -2.138 |
| PRKD1 | protein kinase D1 | Cytoplasm | kinase | -2.089 |
| MX2 | myxovirus (influenza virus) resistance 2 (mouse) | Nucleus | enzyme | -2.081 |
| CSF3 | colony stimulating factor 3 (granulocyte) | Extracellular Space | cytokine | -2.076 |
| AK3L1 | adenylate kinase 3-like 1 | Cytoplasm | kinase | -1.976 |
| ZNF367 | zinc finger protein 367 | Nucleus | transcription regulator | -1.969 |
| SKI | v-ski sarcoma viral oncogene homolog (avian) | Nucleus | other | -1.944 |
| TLX3 | T-cell leukemia homeobox 3 | Nucleus | transcription regulator | -1.943 |
| MYBPH | myosin binding protein H | Cytoplasm | other | -1.935 |
| NEURL2 | neuralized homolog 2 (Drosophila) | Cytoplasm | other | -1.919 |
| ZNF827 | zinc finger protein 827 | Unknown | other | -1.913 |
| NAV2 | neuron navigator 2 | Nucleus | other | -1.898 |
| RGS20 | regulator of G-protein signaling 20 | Cytoplasm | other | -1.887 |
| DFFB | DNA fragmentation factor, 40kDa, beta polypeptide (caspase-activated DNase) | Nucleus | enzyme | -1.882 |
| GPR132 | G protein-coupled receptor 132 | Plasma Membrane | G-protein coupled receptor | -1.838 |
| HIST1H3D | histone cluster 1, H3d | Nucleus | other | -1.837 |
| STMN4 | stathmin-like 4 | Cytoplasm | other | -1.774 |
| SERINC4 | serine incorporator 4 | Unknown | other | -1.746 |
| NMB | neuromedin B | Extracellular Space | other | -1.740 |
| ABCA6 | ATP-binding cassette, sub-family A (ABC1), member 6 | Plasma Membrane | transporter | -1.735 |
| LPAR1 | lysophosphatidic acid receptor 1 | Plasma Membrane | G-protein coupled receptor | -1.716 |
| PDPN | podoplanin | Plasma Membrane | transporter | -1.716 |
| APBA2 | amyloid beta (A4) precursor protein-binding, family A, member 2 | Cytoplasm | transporter | -1.714 |
| BST1 | bone marrow stromal cell antigen 1 | Plasma Membrane | enzyme | -1.705 |
| LPA | lipoprotein, Lp(a) | Extracellular Space | other | -1.699 |
| PLXNA1 | plexin A1 | Plasma Membrane | transmembrane receptor | -1.697 |
| DLX2 | distal-less homeobox 2 | Nucleus | transcription regulator | -1.696 |
| UBR4 | ubiquitin protein ligase E3 component n-recognin 4 | Nucleus | other | -1.696 |
| NKX6-2 | NK6 homeobox 2 | Nucleus | transcription regulator | -1.675 |
| IER3 | immediate early response 3 | Cytoplasm | other | -1.653 |
| C1ORF89 | chromosome 1 open reading frame 89 | Unknown | other | -1.650 |
| GRM2 | glutamate receptor, metabotropic 2 | Plasma Membrane | G-protein coupled receptor | -1.647 |
| AMH | anti-Mullerian hormone | Extracellular Space | growth factor | -1.640 |
| WDR42A | WD repeat domain 42A | Unknown | other | -1.638 |
| SLA | Src-like-adaptor | Plasma Membrane | other | -1.635 |
| GCET2 | germinal center expressed transcript 2 | Unknown | other | -1.624 |
| SOLH | small optic lobes homolog (Drosophila) | Unknown | peptidase | -1.617 |
| ARC | activity-regulated cytoskeleton-associated protein | Cytoplasm | other | -1.615 |
| RPL27A | ribosomal protein L27a | Nucleus | other | -1.607 |
| ADAMTS13 | ADAM metallopeptidase with thrombospondin type 1 motif, 13 | Extracellular Space | peptidase | -1.603 |
| MGA | MAX gene associated | Nucleus | transcription regulator | -1.600 |
|  |  |  |  |  |
| Up-regulated in DOC2/hDAB2 expressing C666-1 cells | |  |  |  |
|  |  |  |  |  |
| Symbol | Entrez Gene Name | Location | Type | Fold Change |
| HNRNPCL1 | heterogeneous nuclear ribonucleoprotein C-like 1 | Nucleus | other | 10.221 |
| WDHD1 | WD repeat and HMG-box DNA binding protein 1 | Nucleus | other | 9.897 |
| ANXA2 | annexin A2 | Plasma Membrane | other | 9.286 |
| RPL22 | ribosomal protein L22 | Nucleus | other | 8.548 |
| ARVCF | armadillo repeat gene deletes in velocardiofacial syndrome | Plasma Membrane | other | 7.384 |
| FKSG17 | nascent-polypeptide-associated complex alpha polypeptide pseudogene 1 | Unknown | other | 7.313 |
| ANO8 | anoctamin 8 | Unknown | other | 6.301 |
| KLF6 | Kruppel-like factor 6 | Nucleus | transcription regulator | 6.182 |
| FLJ11292 | hypothetical protein FLJ11292 | Unknown | other | 5.717 |
| CBWD3 | COBW domain containing 3 | Unknown | other | 5.516 |
| SET | SET nuclear oncogene | Nucleus | phosphatase | 5.509 |
| ICMT | isoprenylcysteine carboxyl methyltransferase | Cytoplasm | enzyme | 5.212 |
| DAGLA | diacylglycerol lipase, alpha | Unknown | other | 4.796 |
| PABPC3 | poly(A) binding protein, cytoplasmic 3 | Cytoplasm | other | 4.537 |
| UACA | uveal autoantigen with coiled-coil domains and ankyrin repeats | Cytoplasm | other | 4.389 |
| NEUROG1 | neurogenin 1 | Nucleus | transcription regulator | 4.259 |
| TBC1D3 | TBC1 domain family, member 3 | Unknown | other | 4.238 |
| MORF4 | mortality factor 4 | Nucleus | transcription regulator | 4.210 |
| DGKH | diacylglycerol kinase, eta | Cytoplasm | kinase | 4.108 |
| TFAM | transcription factor A, mitochondrial | Cytoplasm | transcription regulator | 4.060 |
| IGF1 | insulin-like growth factor 1 (somatomedin C) | Extracellular Space | growth factor | 3.856 |
| HPS1 | Hermansky-Pudlak syndrome 1 | Cytoplasm | other | 3.661 |
| PCLO | piccolo (presynaptic cytomatrix protein) | Cytoplasm | transporter | 3.565 |
| ZBTB20 | zinc finger and BTB domain containing 20 | Nucleus | other | 3.502 |
| CLK3 | CDC-like kinase 3 | Nucleus | kinase | 3.478 |
| STYX | serine/threonine/tyrosine interacting protein | Cytoplasm | phosphatase | 3.415 |
| HIST1H3I | histone cluster 1, H3i | Unknown | other | 3.402 |
| RPL24 | ribosomal protein L24 | Cytoplasm | other | 3.185 |
| CXCL5 | chemokine (C-X-C motif) ligand 5 | Extracellular Space | cytokine | 3.161 |
| PA2G4 | proliferation-associated 2G4, 38kDa | Nucleus | transcription regulator | 3.135 |
| CCNYL1 | cyclin Y-like 1 | Unknown | other | 3.095 |
| HNRNPA1 | heterogeneous nuclear ribonucleoprotein A1 | Nucleus | other | 2.991 |
| SUMO2 | SMT3 suppressor of mif two 3 homolog 2 (S. cerevisiae) | Nucleus | other | 2.956 |
| HNRNPA1L2 | heterogeneous nuclear ribonucleoprotein A1-like 2 | Nucleus | other | 2.927 |
| SULT1A2 | sulfotransferase family, cytosolic, 1A, phenol-preferring, member 2 | Cytoplasm | enzyme | 2.910 |
| GPR44 | G protein-coupled receptor 44 | Plasma Membrane | G-protein coupled receptor | 2.878 |
| WAS | Wiskott-Aldrich syndrome (eczema-thrombocytopenia) | Cytoplasm | other | 2.829 |
| ZNF107 | zinc finger protein 107 | Nucleus | other | 2.824 |
| GAS8 | growth arrest-specific 8 | Cytoplasm | other | 2.808 |
| ST8SIA3 | ST8 alpha-N-acetyl-neuraminide alpha-2,8-sialyltransferase 3 | Cytoplasm | enzyme | 2.777 |
| HIST1H3E | histone cluster 1, H3e | Unknown | other | 2.690 |
| SRP9 | signal recognition particle 9kDa | Cytoplasm | other | 2.635 |
| HPSE | heparanase | Plasma Membrane | enzyme | 2.622 |
| PACRG | PARK2 co-regulated | Cytoplasm | other | 2.618 |
| C12ORF35 | chromosome 12 open reading frame 35 | Unknown | other | 2.597 |
| C9ORF86 | chromosome 9 open reading frame 86 | Unknown | other | 2.561 |
| ELAVL3 | ELAV (embryonic lethal, abnormal vision, Drosophila)-like 3 (Hu antigen C) | Nucleus | other | 2.522 |
| CHMP5 | chromatin modifying protein 5 | Cytoplasm | other | 2.511 |
| SLIT3 | slit homolog 3 (Drosophila) | Extracellular Space | other | 2.508 |
| LOC389842 | similar to RanBP1 | Cytoplasm | other | 2.455 |

Additional file 2 Table S2 Expression changes in C666-1 cells transfected with DOC2/hDAB2. *Values are QRT-PCR mean fold change for triplicates of DOC2/hDAB2 transfected C666-1 cells compared to vector control cells.

| Gene symbol | Accession No. | Microarray | QRT-PCR* |
| --- | --- | --- | --- |
| AKT1 | NM_5163 | -1.27 | -1.09 |
| APC2 | NM_5883 | -1.40 | -2.13 |
| CTRL | NM_1907 | -1.60 | -1.79 |
| MAP4K5 | NM_198794 | 1.50 | 1.79 |
| MERTK | U08023 | -1.50 | -1.91 |
| PPP2R2C | NM_20416 | 1.78 | 1.39 |
| TFAM | NM_3201 | 4.00 | 1.09 |
| TFF-1 | NM_3225 | -1.50 | -2.39 |
| TRAF7 | NM_32271 | 1.70 | 1.08 |
| CSF3 | NM_759 | -2.00 | -12.24 |
| MX2 | NM_2463 | -2.00 | 1.02 |
| SUMO2 | NM_6937 | 3.00 | 1.62 |
| FOS | NM_5252 | -1.15 | -1.12 |
| TGFB1 | NM_660 | -1.26 | -1.45 |
